# Relating to AI in Spiritual Care

**DOI:** 10.2196/113467

**Published:** 2026-11-09

**Authors:** Fransisca Christiane Warmenhoven, Bauk Wilhelmina Jan Aartsen, Roos Notermans

**Affiliations:** 1 Department of Spiritual Care, Maastricht University Medical Centre, P. Debyelaan 25, Maastricht, Limburg, 6229HX, The Netherlands, +31(0) 43-3875338

**Keywords:** AI, chatbots, spirituality, spiritual care, existential crisis, consciousness, relationship, Martin Buber

## Abstract

In response to a recently published Delphi study on AI in spiritual care, this commentary focuses on ways to perceive the relationship between AI and human beings in the context of spiritual care. We explore this relationship by framing it within the philosophy of Martin Buber. Because AI has no lived experience or existential crises of its own, we question whether a truly fulfilling encounter between a human being and an AI model is possible when the relationship is inherently asymmetrical on an existential level.

## Introduction

With interest, we read the article “Artificial Intelligence in Spiritual Care: Modified Delphi Study,” in which Winiger et al [1] present an international multistakeholder Delphi study to investigate the use of AI in spiritual care. We applaud the effort that was made to bring awareness to the use of AI in spiritual care. It is essential for us, as health care professionals in spiritual care, to engage with AI, not only because developments in AI are happening at a rapid pace but also because the general public are already making widespread use of AI.

In their Delphi study, Winiger et al [1] report that the panel agreed most strongly on the ability of AI to engage in administrative and informational tasks and that relational tasks remain understood as a primarily human responsibility. However, as the use of AI is becoming more widespread, people are turning to AI with emotional and existential issues of their own accord. A recent article by Cole-Turner [2] reports that participants who shared various aspects of their spiritual lives with a chatbot experienced the AI tool as a guide and companion in their spiritual journey. The use of AI in spiritual care is thus not merely dependent on how spiritual caregivers position AI in their professional field. People now, and probably more frequently in the future, will turn to AI as a conversational partner on topics including existential issues.

## We Performed a Small Experiment

In a small study [3] in December 2025, we conducted a Dutch-language experiment using 4 AI models: Gemini 3 Pro Preview (Google), Claude Sonnet 4.5 (Anthropic), Grok 4 (SpaceXAI), and GPT-5 (OpenAI). We presented a common chaplaincy scenario in which a person is keeping vigil at the bedside of their dying father and is at a loss what to do or how to be in this situation. When explicitly prompted to act as a spiritual care provider, the AI models performed relatively well at providing information, normalizing emotions, and offering reassurance. They offered metaphors (“A lot of people feel overwhelmed in a moment like this—it’s like a storm in which everything familiar seems to shake“ [Grok 4]), named ambivalence (“This must be so heavy for you, it’s beautiful but at the same time so difficult“ [Claude Sonnet 4.5]) and simulated nonverbal communication (“I move a little closer and take my time before I reply“ [Claude Sonnet 4.5])

Additionally, we assumed (but did not test) that AI would surpass many spiritual caregivers in its ability to readily use hermeneutic sources from different philosophical and religious contexts. However, we perceived several limitations in the use of AI in spiritual care. First, AI models, because of their programming, cannot help working from the perspective that even existential issues are problems to be solved instead of experiences to be lived and shared in relationship with other beings. Second, despite their access to almost limitless hermeneutic sources, AI models do not have the hermeneutic competence to bring the right source in the right moment to the right person. And finally, and perhaps most importantly, AI models do not have lived experiences of existential issues that they can bring into the relationship. The AI chatbots differ on a fundamental level from human beings: human beings are mortal beings, and even more so, they can be conscious of their mortality [4].

## Conclusion

According to the Delphi study, the loss of human connection was the highest-ranked risk in the use of AI in spiritual care [1]. Apparently, the human connection or the relationship between two human beings is seen as essential in spiritual care. Drawing on Martin Buber’s philosophy [5], the relationship depends on whether one can establish an I-thou relationship with another being instead of an I-it relationship. The I-thou relationship can lead to a sense of closeness to the other, and to oneself, and enables the beings that are part of the relationship to come into each other’s full presence. It even opens the connection to the transcendental. In Buber’s philosophy, this I-thou relationship is not merely reserved for pairs of human beings, but can also exist between a human being and an animal or another natural being, such as a tree. Buber argues that the I-thou relationship is determined by how human beings choose to relate to each other. Therefore, we end this commentary with a question: is it possible for a human being to have an I-thou relationship with an AI? Many questions will follow from this exploration, and we can only hope that research, with the help of AI, will be able to catch up with the speed of current developments.
